# Periodically Self‐Pulsating Microcapsule as Programmed Microseparator via ATP‐Regulated Energy Dissipation

**DOI:** 10.1002/advs.201700591

**Published:** 2018-01-04

**Authors:** Xiang Hao, Liang Chen, Wei Sang, Qiang Yan

**Affiliations:** ^1^ State Key Laboratory of Molecular Engineering of Polymers Fudan University Shanghai 200433 China

**Keywords:** dissipative self‐assembly, microseparators, self‐adaptive polymers, smart vesicles

## Abstract

Living systems can experience time‐dependent dynamic self‐assembly for periodic, adaptive behavior via energy dissipation pathway. Creating in vitro mimics is a daunting mission. Here a “living” giant vesicle system that can perform a periodic pulsating motion using adenosine‐5'‐triphosphate (ATP)‐fuelled dissipative self‐assembly is described. This dynamic system is built on transient supramolecular interactions between the polymer and cellular energy currency ATP. The vesicles capturing ATPs will deviate away from equilibrium, leading to an energy ascent that drives a continuous vesicular expansion, until a competitive ATP hydrolysis predominates to break the ATP–polymer interactions and deplete the energy stored in the vesicles, leading to an opposing vesicular contraction. The input of ATP energy can sustain that these vesicles run periodically along this reciprocating expansile–contractile process, resembling a “pulsating” behavior. ATP level can orchestrate the rhythm, amplitude, and lifetime of this biomimetic pulsation. By pre‐programming the ATP stimulation protocol, this kind of adaptive microcapsules can function as high‐performance microseparators to perform size‐selective sieving of different nanoparticles through ATP‐mediated transmembrane traffic. This man‐made system offers a primitive model of time‐dependent dynamic self‐assembly and may offer new ways to build life‐like materials with biomimetic functions.

## 1 Introduction

Energy dissipation, as one of the most inherent attribute of living systems, underpins biologically dynamic and periodic behaviors.^1^ For example, biological cells use the chemical fuel, adenosine‐5'‐triphosphate (ATP), to dynamically regulate cell activity and viability;^2^ microtubules sustain their switchable lengthening/shortening by consuming the energy from guanosine triphosphate.^3^, ^4^ These naturally occurring dissipative systems have been known very early; however, in vitro mimicking such “living” systems with adaptive features using “nonliving” molecular building blocks is still a fundamental challenge in chemistry.^5^, ^6^

To date, chemists have established a plethora of synthetic self‐assembly entities,^7^ some of which can undergo reversible shape changes or phase transitions under external stimuli,^8^, ^9^, ^10^ but they are not yet comparable to their natural counterparts. The main reason is that synthetic self‐assembly systems operate in thermodynamic equilibrium, resulting in time‐independent steady‐state structures. In contrast, natural self‐assembly requires a continuous supply of energy to keep the assemblies away from equilibrium, resulting in time‐dependent dynamic structures.^11^ If the energy dissipates, the system will fall apart and revert to ground state. This process is referred to as *dissipative self‐assembly*,^12^ which offers impetus for biological motions such as cell deformation and growth (in situ motion without distance change), and cell migration and division (motion with distance change). Hence, implanting this energy‐driven assembly in man‐made assemblies may create life‐like systems, possessing structural adaption and behavioral evolution over time and space. However, such artificial systems formed by dissipative pathway are quite limited thus far. The few reports focused on biocatalytic active gels,^13^, ^14^, ^15^ switchable films,^16^ and molecule‐level reaction networks.^17^ Nanoscale nonequilibrium assemblies have received little attention. An insuperable obstacle is how to harmonize sophisticated self‐assembly process with continuous energy influx in a time‐ordered manner.

Herein, we report a “living” giant vesicle system that can perform autonomous and periodic pulsating motion via energy dissipation. The energy source of this pulsation derives from the cellular biochemical fuel, ATP. To achieve the energy‐regulated self‐assembly, our design concept is based on transient ligand–receptor supramolecular interactions between the ATP fuels and the vesicular membrane components. The system is necessary to satisfy the following conditions: (i) a self‐assembling vesicle with specific ATP receptor units can toggle between expansile and contractile state by the association and dissociation of ATP; (ii) the temporary formation of ATP–polymer ligand–receptor complexes can cause the system energy gain far from equilibrium, thus activating a forward vesicular expansion; (iii) a competing effect for breaking these complexes can dissipate the energy stored in the system, leading to a backward vesicular contraction to initial state; (iv) two different pathways of activating and deactivating the noncovalent interactions can constitute a feedback close‐loop for lasting the action of sacs. As long as supplying continuous ATP energy, this reversible expansion–contraction process of the vesicles, like cellular pulsation, will periodically and autonomously proceed over time.

## 2 Results and Discussion

### 2.1 Polymer Design and Vesicular Self‐Assembly

**Figure 1:**
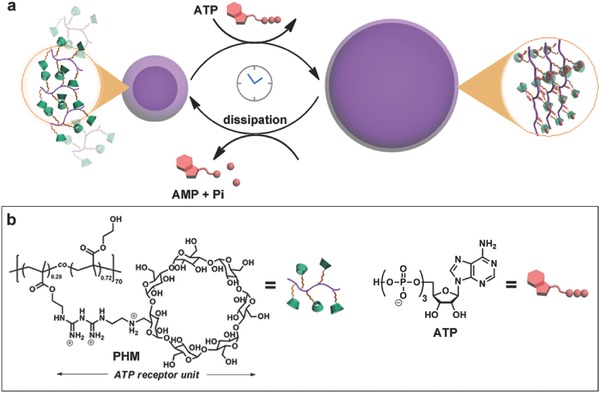
a) Schematic illustration of the periodically pulsating polymer vesicular system driven by cyclical association/dissociation of ATP–polymer complex through dissipative self‐assembly process. b) The chemical structure of ATP receptor‐appended polymer (PHM) and ATP fuel.

To meet the above criteria, we design and fabricate a class of giant polymer vesicles with switchable phase behaviors. The polymer is made up of commercial poly(2‐hydroxyethyl methacrylate) homopolymer, randomly modified by pocket‐shaped ATP receptor as the side group (termed as PHM, per chain has ≈20 receptors, in **Figure** **1**b). Each receptor unit has one β‐cyclodextrin host and a positively charged biguanidine spacer, which is previously demonstrated to be capable of efficiently trapping ATP molecule by specific ligand–receptor interactions.^18^ Fuelled by ATP, we anticipated that the polymersomes would deviate away from the thermal equilibrium via capture of ATP. This may offer an energetic uphill path to drive an expansion of vesicular membranes. For the competing path, we utilize potato apyrase, a hydrolase that can decompose ATP, to break the ATP–polymer interactions.^19^ This may favor the energy release and thus lead to a reverse vesicular contraction. The synchronous introduce of ATP and enzyme sets up a transient supramolecular cycle, rendering the system to autonomously run along between the equilibrium and nonequilibrium state, in order to fulfil the reciprocating motion of vesicular membrane (Figure 1a).

**Figure 2:**
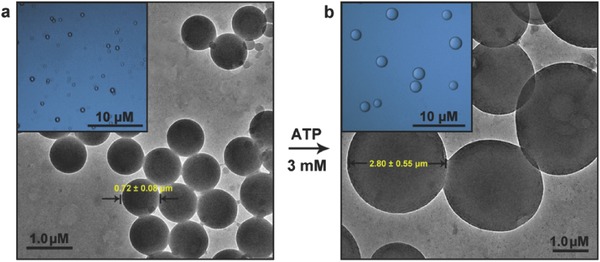
a) TEM and bright‐field optical image (inset) of initial PHM vesicles in aqueous solution without any stimulus (*C*_PHM_ = 150 × 10^−6^ m). b) TEM and bright‐field optical image (inset) of the membrane extension of the PHM vesicles after treatment with 3.0 × 10^−3^ m of ATP during one hour incubation.

We first investigated the vesicular swelling process under ATP trigger only. Owing to the hydrophilic side groups and hydrophobic main chain coexisting in the PHM chain, they can spontaneously form aggregates in aqueous media (polymer concentration, *C*_PHM_ = 150 × 10^−6^ m). transmission electron microscopy (TEM) image displayed that the aggregates have large spherical morphology with the size of 0.72 ± 0.08 µm (**Figure** **2**a). Optical microscope further revealed that these spheres are hollow vesicles, as characterized by the contrast between the gray edge and bright central part in each object (Figure 2a, inset). Upon the addition of ATP (3.0 × 10^−3^ m), interestingly, the vesicles produced a self‐assembled transition. Their eventual scales appeared nearly fourfold increase up to 2.80 ± 0.55 µm, while their globular structure kept constant (Figure 2b and inset). Particulate autostatistics from the optical microscope and laser light scattering (LLS) results also confirmed this ATP‐induced vesicular growth averagely from 0.77 to 2.91 µm (Figures S1 and S2, Supporting Information), consistent with the TEM images. LLS further showed that this growth is continuous, and the swelling rate averages at ≈90 nm min^−1^ (Figure S3, Supporting Information). Because ATP can bind with the receptor units in the polymer, a plausible reason for this arises from an increment of membrane hydrophobicity as a result of the ATP–polymer polyplex formed. To validate this, an apolar‐sensitive probe, 1,6‐diphenyl‐1,3,5‐hexatriene (DPH, λ_em_ = 428 nm), was anchored on the vesicular wall to detect the change of hydrophobic region.^20^ As expected, the fluorescence produced a dramatic jump over time (Figure S4, Supporting Information), verifying our viewpoint on ATP‐induced hydrophobic change. In addition, noting that both the initial and final sacs are stable over two weeks without fission or fusion, suggesting that this expansion is indeed attributed to the membrane extension of single microcapsule (Figure S5, Supporting Information).

### 2.2 ATP‐Fuelled Periodic Pulsation of Giant Polymeric Vesicles

**Figure 3:**
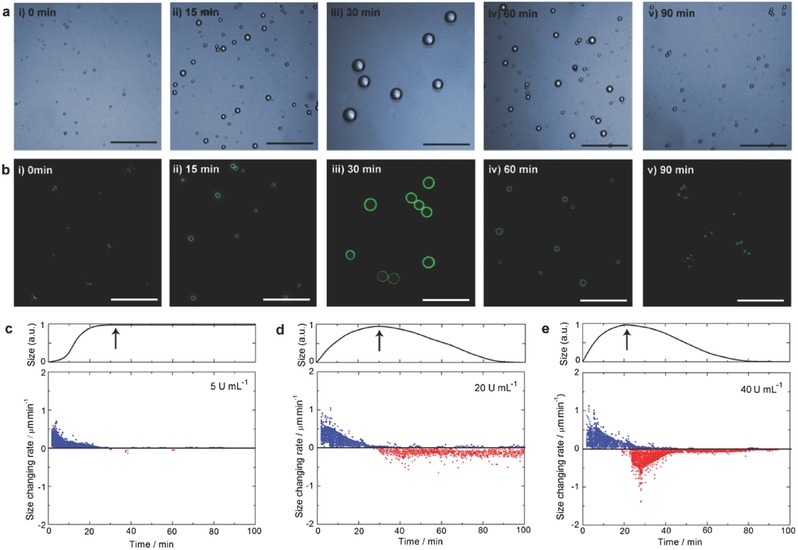
a) Optical and b) confocal microscope tracking the autonomous vesicular membrane motion within one cycle: i–iii) the forward vesicular expansion in the first regime (from 0 to 30 min); iii–v) the backward vesicular contraction in the second regime (from 30 to 90 min). The vesicle solution is exerted to 3.0 × 10^−3^ m of ATP fuel under pretreatment with potato apyrase (20 U mL^−1^). DPH fluorescent probe (1 × 10^−5^ m) is anchored onto the vesicle membrane for testing the change of polymer hydrophobicity. c–e) Upper panel: normalized vesicle size change against time under different enzyme levels. The arrows indicate the moments that the vesicles flip‐flop their membrane moving direction. Lower panel: rates of forward vesicle growth (blue dot) and backward vesicle shrinkage (red dot) at various enzyme levels, c), 5 U mL^−1^; d), 20 U mL^−1^; e), 40 U mL^−1^ over time. Scale bars are 10 µm.

After confirming the forward vesicular expansion driven by ATP–polymer supramolecular recognition, we desired that this process can automatically return back. To fulfil this goal, a competing path to deplete the chemical energy stored in the polymer membranes is necessitated. Potato apyrase is used as a proper candidate for this since it can hydrolyse ATP to adenosine‐5′‐monophosphate (AMP) and two phosphoric acid (Pi) species. ATP favors to bind our polymer with high affinity (*K*_B_ = 7.5 × 10^7^ m ^−1^);^18^ however, neither AMP nor Pi can form complexes with the receptor unit in polymer (*K*_B_ < 10^2^ m ^−1^; Table S1, Supporting Information). Hence, once the ATP is enzymatically decomposed, the binding energy stored in the polymeric membrane would be increasingly release. Based on this, we exerted ATP (3.0 × 10^−3^ m) to the vesicular system under pretreatment with this hydrolase (20 U mL^−1^) and monitored their self‐assembly in real‐time. Bright‐field optical images showed that the evolutionary process can be divided into two distinct regimes: in the first regime (0→30 min), all the polymersomes self‐dilated increasingly from the initial size of 0.72 ± 0.08 µm to a maximum value of 2.65 ± 0.40 µm (**Figure** **3**a(i–iii)); in the second regime, they went through a self‐shrinkage back to their initial size (0.75 ± 0.12 µm) but spending a longer time around 60 min (Figure 3a(iii–v)). If the vesicles loaded the DPH fluorescent probes, this reversible expansion–contraction can be further evidenced by laser confocal microscopy (Figure 3b(i–v)). Moreover, accompanying with the variation trend of vesicle volume, their fluorescence intensity appeared a coincident change as well (Figure S6, Supporting Information). These results mean that there is a supramolecular cycle inside this system: first, ATP fuelled the formation of polyplex to increase the hydrophobicity, leading to the vesicle expansion; subsequently, the process of ATP consumption takes over the system in turn, the vesicle contraction occurs due to the decrease of systemic hydrophobicity.

To gain deep insight into the relationship between the time scales of vesicular evolution and the underlying supramolecular cycles, we investigated the evolutionary dynamics of the vesicles under different enzyme levels using confocal microscope. In all cases, once addition of ATP fuel the vesicle expansion could be observed (upper panels in Figure 3c–e). At low ATP hydrolytic rate (enzyme level = 5 U mL^−1^), the vesicle size gradually increased in the first 32 min then levelled off (Figure 3c, upper panel), but the drop of sizes were barely seized (Figure 3c, lower panel). With the acceleration of hydrolysis (enzyme level rises to 20 and 40 U mL^−1^), the vesicles exhibited more rapid expansion and reached maximum sizes in shorter periods (30 and 21 min), after which they entered a shrinking regime and recovered their initial states (Figure 3d,e). The transition between the two regimes coincided well with the moment the vesicle membranes switched their moving direction. More importantly, the run cycle of the vesicular expansion–contraction depends on the enzyme level, which potentially becomes a basis to regulate their periodic behavior.

**Figure 4:**
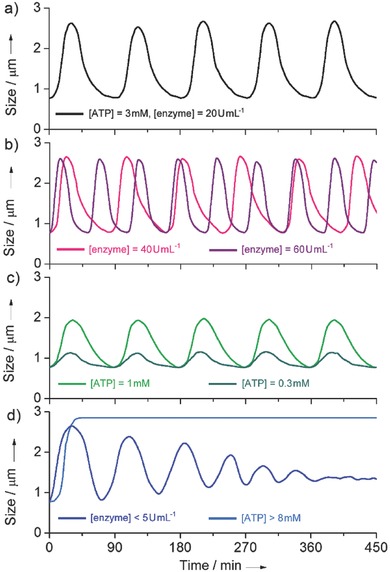
a) A standard vesicle pulsation showing ≈90 min periodicity and fourfold changing amplitude (*C*_ATP_ = 3.0 × 10^−3^ m and *C*_enzyme_ = 20 U mL^−1^). b) Enzyme‐dependent pulsating periodicity adjustability: *C*_enzyme_ = 40 U mL^−1^ (pink curve, 82 min periodicity) and *C*_enzyme_ = 60 U mL^−1^ (purple curve, 64 min periodicity). The ATP levels are fixed at 3.0 × 10^−3^ m. c) ATP‐dependent pulsating amplitude adjustability: *C*_ATP_ = 1.0 × 10^−3^ m (green curve, 2.5‐fold size change) and *C*_ATP_ = 0.3 × 10^−3^ m (olive curve, 1.6‐fold size change). The enzyme levels are fixed at 20 U mL^−1^. d) The cases of vesicle pulsating decay (blue curve) and termination (navy curve) when enzyme level is less than 5 U mL^−1^ and when ATP level is over 8.0 × 10^−3^ m.

If we defined this vesicular expansion–contraction as one motion cycle, we wanted to know whether this process can be re‐initiated. LLS was used to track the vesicular change under different conditions over time. Incubation of potato apyrase with 20 U mL^−1^, triggered by ATP (3.0 × 10^−3^ m), the vesicles first grew until climbing up to a size extreme (2.73 ± 0.62 µm), then followed by an automatic decay back to the beginning size, which is in line with the above optical results. But strikingly, when the system recovered, a new cycle could be refuelled. This process can automatically persist over 15 cycles without decay, and the size change plotted against time is analogous to a sinusoidal wave fashion (**Figure** **4**a, only shows 5 cycles), as if the vesicles do reciprocating movement in a period of ≈90 min. As compared to other dissipative systems,^13^, ^14^, ^15^ the lifetime of our vesicles is much longer (exceeds ≈1400 min) and the phase transitional period shows accurate time dependence. A reasonable reason is that the generated chemicals (AMP and Pi) have no negative effect on the systemic self‐assembly process. Fluorescence recording corroborated this oscillating‐like behavior through DPH‐loaded vesicles (Figure S7, Supporting Information). This ATP‐fuelled periodic vesicular expansive–contractive process is in many ways reminiscent of cellular “pulsating” feature. The two pulsating extreme (size minimum and maximum) can be identified as the steady‐state assemblies in thermal equilibrium and the transient‐state assembling structure far from equilibrium, respectively, where the system periodically runs along between the two states.

In an effort to tune the periodicity and amplitude of this vesicular pulsating process, we varied the concentration of ATP fuel and internalized enzyme. When the enzyme level increased from 20 to 40 and 60 U mL^−1^ but the ATP level kept constant at 3.0 × 10^−3^ m, a remarkable pulsating acceleration (periodicity shortened from 90 to 82 and 64 min, respectively) appeared while their size pulsating scope was unaffected (Figure 4b). On the contrary, if the enzyme level was fixed but lowering the ATP level from 3.0 × 10^−3^ to 1.0 × 10^−3^ and 0.3 × 10^−3^ m, the pulsating scope depressed correspondingly (size jump from 4.0‐fold to 2.5‐fold and 1.6‐fold, respectively) whereas their periodicity only had neglected changes (Figure 4c). These results indicate that the frequency and amplitude, the two physical parameters to describe this vesicular pulsating behavior, is positively correlated to the dose of the enzyme and ATP, respectively (Figures S8 and S9, Supporting Information). In addition, we found if the enzyme was below 5 U mL^−1^ or ATP was above 8.0 × 10^−3^ m, a pulsating attenuation or termination would appear (Figure 4d), indicating that there exists optimal conditions to sustain this dynamic self‐assembly evolution (*C*_ATP_ < 8.0 × 10^−3^ m and *C*_enzyme_ > 5 U mL^−1^).

### 2.3 Vesicular Self‐Pulsating Mechanism

Insight into the transient supramolecular cycle governing this dynamic system was preliminarily verified by ^31^P NMR monitoring (Figure S10, Supporting Information). ATP alone had three characteristic phosphorus signals at −9.5 (γ‐P), −10.9 (α‐P), and −21.3 (β‐P) ppm. During the forward vesicular expansion, these three signals shifted to downfield accompanying with spin–spin splitting to −7.5 (d, ^3^ *J*_β,γ_ = 11.4 Hz), −8.6 (d, ^3^ *J*_α,β_ = 16.5 Hz), and −19.3 ppm, respectively, indicating the complexation of ATP and the polymer. While the vesicles backward shrink, two new peaks at −6.3 and +1.2 ppm ascribed to AMP and Pi species enhanced, which is indicative of the dissociation of the complex. This reversible ligand–receptor chemistry could be further supported by UV–vis (Figure S11, Supporting Information).

**Figure 5:**
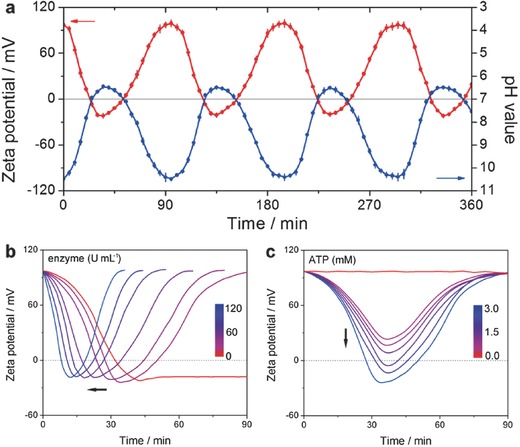
a) The oscillating change of vesicle surface zeta potential (blue curve) and solution pH (red curve) as a function of vesicle pulsating time. *C*_enzyme_ = 20 U mL^−1^, *C*_ATP_ = 3.0 × 10^−3^ m (ATP is refuelled the system once the zeta potential climbs up to the wave peak). Zeta potential analysis revealing that the enzyme or ATP level adjusts the periodicity and amplitude of the vesicle pulsation (monitoring in one cycle): b) *C*_enzyme_ linearly varying: 0, 20, 40, 60, 80, 100, and 120 U mL^−1^ (from right to left, *C*_ATP_ is fixed at 3.0 × 10^−3^ m); c) *C*_ATP_ linearly varying: 0, 0.5, 1.0, 1.5, 2.0, 2.5, and 3.0 × 10^−3^ m (from top to down, *C*_enzyme_ is fixed at 20 U mL^−1^.

According to the above results, we proposed a possible energy dissipation mechanism to explain this vesicle pulsation, as depicted by Figure 1a: (i) without ATP, the whole system is in equilibrium and there are many positively charged receptors distributed on the vesicle membrane. (ii) Upon addition of negatively charged ATP, they can specifically bind with the membrane receptors. The charge neutralization enlarges the hydrophobicity of the membranes, which renders the system to increasingly deviate away from equilibrium; as a result, to compensate the increased free energy, a continuous vesicular expansion will be activated. The vesicles do not stop to grow until the ATP fuels use up. (iii) Since the competing enzymatic reaction path can reversibly destruct the supramolecular interactions, the surface charge can recover in order to release the energy stored in the vesicles, which leads to an autonomous self‐assembly membrane contraction. To validate this assumption, zeta potential experiments offered key evidence on the variation of membrane potential (**Figure** **5**a). For the case of treatment with 3.0 × 10^−3^ m of ATP and 20 U mL^−1^ enzyme, identical to the periodic evolution of vesicular expansion–contraction, the zeta potential also switched from +97 mV to −21 mV (Figure 5a, red curve), accompanied by the solution pH fluctuation from weak alkalinity of 10.48 to near neutrality of 6.45 (Figure 5a, blue curve), confirming the oscillating variation of the assemblies between electropositivity and electroneutrality. Furthermore, the potential and pH changing periodicity is evaluated to be 95 min concordant with that pulsating period (90 min), which demonstrates that the mechanism of transient supramolecular cycle underpinning the periodic pulsation of polymer vesicles is reasonable.

In addition, we attempted to explain the tunability of vesicle pulsating parameters (periodicity and amplitude). On the one hand, increasing the enzyme concentration (from 0 to 120 U mL^−1^ with an interval of 20 U mL^−1^) can highly enhance the rate of backward dissociation between ATP and the polymer (Figure S12, Supporting Information). Thus, the pulsating periodicity can be shortened correspondingly (0 U mL^−1^: +∞; 20 U mL^−1^: 95 min; 40 U mL^−1^: 81 min; 60 U mL^−1^: 66 min; 80 U mL^−1^: 53 min; 100 U mL^−1^: 45 min, and 120 U mL^−1^: 36 min, respectively), which is reflected by the accelerated recovery of zeta potential with the increase of enzyme concentration (Figure 5b). On the other hand, increasing the ATP concentration (from 0 to 3.0 × 10^−3^ m with an interval of 0.5 × 10^−3^ m) can strongly affect the degree of forward association between ATP and the polymer, which dictates the ATP‐dependent pulsating amplitude (Figure S13, Supporting Information). Corresponding evidence also observed in the zeta potential experiment in which their wave valley values showed an ATP‐dependent descending trend (0 × 10^−3^ m ATP: +94.6 mV; 0.5 × 10^−3^ m ATP: +21.0 mV; 1.0 × 10^−3^ m ATP: +14.2 mV; 1.5 × 10^−3^ m ATP: +6.7 mV; 2.0 × 10^−3^ m ATP: −8.2 mV; 2.5 × 10^−3^ m ATP: −15.6 mV, and 3.0 × 10^−3^ m ATP: −24.5 mV), indicating the incremental degree of ATP/polymer association (Figure 5c). The results of pulsating tunability characterized by zeta potential agree well with that of LLS analysis (Figures S8 and S9, Supporting Information).

### 2.4 ATP‐Regulated Vesicle Transmembrane Traffic as Programmed Microseparators

**Figure 6:**
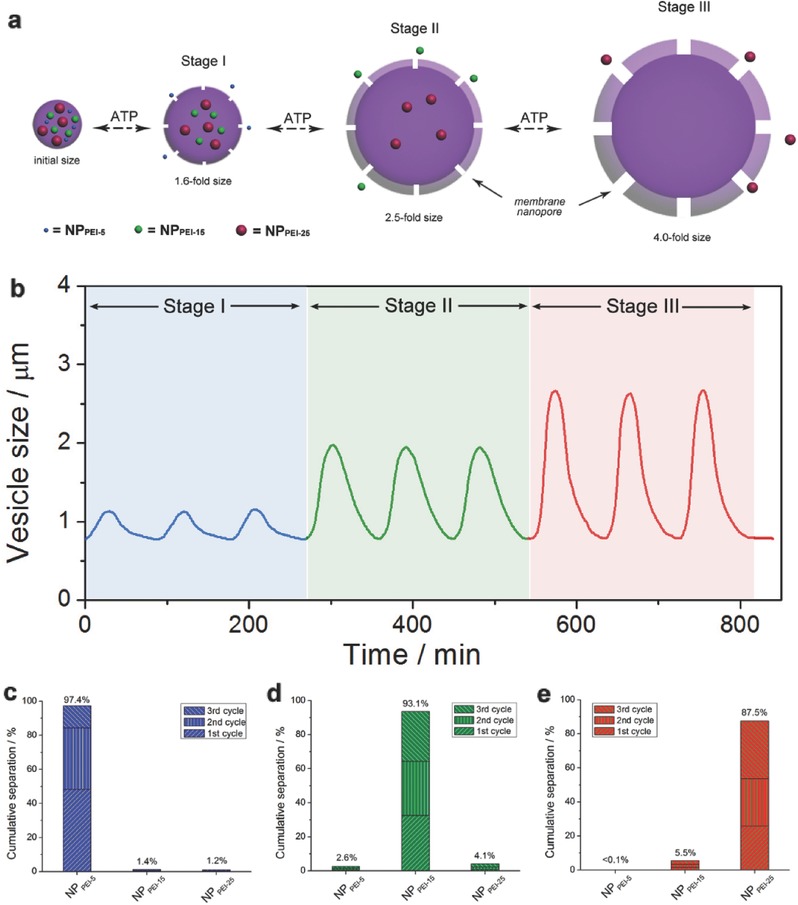
a) Schematic illustration and b) practical size oscillation of programmed vesicle pulsation at three distinct stages (each stage lasts three cycles). The vesicles encapsulated three different sizes of dye‐labeled PEI nanoparticles (NP_PEI‐5_, NP_PEI‐15_, and NP_PEI‐25_). The separation efficiency with respect to the different sizes of nanoparticles in the three stages: c) Stage I, d) Stage II, and e) Stage III.

It is well‐recognized that cell membrane is biologically semipermeable boundary capable of implementing high‐selective substance exchange. Since it has been demonstrated that our vesicles can proceed ATP‐fuelled pulsating‐like membrane movement, we expected that the membrane possesses ATP‐regulated membrane permeability. To elucidate this, we utilized three kinds of hyperbranched poly(ethylene imine) nanoparticles (NP_PEI_) with different nanoscales (NP_PEI‐5_, 4 nm; NP_PEI‐15_, 7 nm; NP_PEI‐25_, 10 nm) to study their selective sieving by the vesicles. Three types of fluorophores (coumarin‐343, rhodamine‐B, and cyanine‐5.5; Figure S14, Supporting Information) were respectively selected to functionalize at the periphery of the three hyperbranched polymers, affording three kinds of dye‐labeled water‐soluble NP_PEI_s with distinct fluorescent emission (NP_PEI‐5_, λ_em_ = 464 nm; NP_PEI‐15_, λ_em_ = 565 nm; NP_PEI‐25_, λ_em_ = 707 nm). Loaded them together in the vesicular lumen, fluorescent microscope was utilized to reveal the membrane‐selective particulate sieving under various ATP trigger conditions (**Figure** **6**a). To realize high‐efficient separation, we designed a programmed stimulation mode: using 0.3 × 10^−3^, 1.0 × 10^−3^, and 3.0 × 10^−3^ m of ATP treated with the above NP_PEI_‐loaded microcapsules, respectively, and each ATP condition was applied to trigger three pulsating cycles of the assemblies. LLS monitoring pointed out that the NP_PEI_‐loaded microcapsules can accordingly experience a programmable membrane pulsation featuring ATP‐dependent size jump scope (Figure 6b).

Interestingly, during the first three cycles (Stage I), accompanying every vesicular pulsating process with the size change from 0.77 to 1.25 µm (1.6‐fold size jump), part of fluorescent nanoparticles might be pumped out and the cumulative release amount of the smallest NP_PEI‐5_ statistically reached up to 97.4% (1st cycle: 48.2% release; 2nd cycle: 36.2% release; 3rd cycle: 13.0% release); in contrast, the NP_PEI‐15_ and NP_PEI‐25_ access appeared to be forbidden by the microcapsule membrane (less than 1.5% escape; Figure 6c). That is to say, when the vesicles have a minor pulsation, the distensible membrane opens some membrane nanopores which can only allow the passage of smallest nanoparticles (4 nm), but block the larger ones. In the Stage II, the amplitude of vesicle pulsation was reinforced (from 0.77 to 1.96 µm, 2.5‐fold size jump), which leads to a size extension of these membrane nanopores. Thus, it caused that almost all of the NP_PEI‐15_ cargos (1st cycle 32.4% + 2nd cycle 32.0% + 3rd cycle 29.4% = total release 93.8%) and the NP_PEI‐5_ residues penetrate across the membrane whereas the largest NP_PEI‐25_ still stayed inside (Figure 6d). Finally, in the Stage III, because the membrane pores reached a maximum value, it allowed the NP_PEI‐25_ to escape (total release 87.5%; Figure 6e).

This is actually analogous to a step‐by‐step extractive process and the pulsating microcapsules can be viewed as high‐selective microseparators. In laboratory, we usually adopt the gradient centrifugal method to separate nanoparticles with different sizes, but this method is subjected to the insufficient separating efficiency and inaccurate size screening, and only be suitable for solid nanoparticles. In comparison, for our microcapsules, they can apply to soft nanoparticles and the separation factor (α) of one kind of nanoparticles with respect to the other two can attain to ≈95, ≈93, and ≈87, respectively, and that the size resolution is less than 3 nm, which is superior to the tradition separation methods. Moreover, fine regulating the ATP dosage or increasing the cyclic number can further improve the separating efficiency and size selectivity. Besides soft nanoparticle, this smart microseparator is applicable to other significant size‐selective substance separation such as mixed inorganic nanoparticles (such as silica NPs; Figure S15, Supporting Information) and multiple protein NP blend (Figure S16, Supporting Information).

## 3 Conclusions

In conclusion, we develop a self‐pulsating vesicular system for the first time that can dissipate chemical energy to maintain their periodic membrane movement in out‐of‐equilibrium state. The transient supramolecular cycle established between ATP and the polymer membrane becomes a driving force to realize the vesicular dynamic evolution. ATP, as a continuous source of chemical fuel, can fine regulate the critical parameters of vesicle pulsation including pulsating periodicity, pulsating amplitude, and operating lifetime. This ATP‐fuelled dissipative self‐assembly pathway represents an entirely new strategy to fulfil the goal of biological motion mimics. The dynamic assemblies are promising to apply as high‐performance microseparator for size‐selective nanoparticle sieving through programmed ATP triggers. We anticipate that these self‐pulsating microcapsules will exemplify the new opportunities of the life‐like assemblies using complex artificial molecular system, and provide new visions from time‐independent static self‐assembly toward time‐ordered dynamic self‐assembly with “living” natures, endowing the system with cell mimicry function.

## Conflict of Interest

The authors declare no conflict of interest.

## Supporting information

Supplementary
