# Supplementary material for: Periodically Self‐Pulsating Microcapsule as Programmed Microseparator via ATP‐Regulated Energy Dissipation
Source: Adv Sci (Weinh). 2018 Jan 4;5(3):1700591. doi: 10.1002/advs.201700591 (PMC5867064; doi:10.1002/advs.201700591)
Supplement: Supplementary file 1 — Supplementary [file ADVS-5-1700591-s001.pdf]

Copyright WILEY-VCH Verlag GmbH & Co. KGaA, 69469 Weinheim, Germany, 2018.

ADVANCED SCIENCE
Open Access

Supporting Information

for *Adv. Sci.,* DOI: 10.1002/advs.201700591

Periodically Self-Pulsating Microcapsule as Programmed Microseparator via ATP-Regulated Energy Dissipation

*Xiang Hao, Liang Chen, Wei Sang, and Qiang Yan**

Copyright WILEY-VCH Verlag GmbH & Co. KGaA, 69469 Weinheim, Germany, 2016.

Supporting Information

Periodically Self-Pulsating Microcapsule as Programmed Micro-separator via ATP-Regulated Energy Dissipation

*Xiang Hao, Liang Chen, Wei Sang, and Qiang Yan**

1. Materials and Methods

Materials. All chemicals were used as received unless indicated. Poly(2-hydroxyethyl methacrylate) ($PHEMA_{70}$, M_w = 9.10 kDa, M_w/M_n = 1.04) was purchased from Polym. Int. and removed the inhibitor by reduced distillation before use. The functional ATP-receptor unit, β-cyclodextrin-modified biguanidine molecule was obtained according to our previous literature[9] for conjugation with the homopolymer PHEMA. Adenosine 5'-monophosphate monohydrate ($AMP{\cdot}H_2O$), adenosine 5'-triphosphoate disodium salt (ATP), and phosphorous acid (Pi) were purchased from Sigma-Aldrich and dissolved in 4-(2-hydroxyethyl)-1-piperazineethane sulfonic acid buffer (HEPES, pH = 7.2~7.4) for use. Apolar-sensitive fluorescent probe, 1,6-diphenyl-1,3,5-hexatriene (DPH, λ_{em} = 428 nm), was purchased from TCI and dissolved in acetone to obtain a stock solution (1.0 mM). The recombinant enzyme, potato apyrase, expressed in Pichia pastoris, was obtained from BioChem. Co. and dissolved in milliQ water to prepare a concentration of 100 U/L working aliquots and preserved at -20 ^{o}C before use.

Preparation of ATP-receptor grafted copolymer PHM. $PHEMA_{70}$ homopolymer (0.455 g, 50 μM), N-hydroxylsuccinimide (0.173 g, 1.5 mM, 30 equiv. to active hydroxyl group) was dissolved in 150 mL of anhydrous dimethylacetamide in round bottom flask, and then added 1-ethyl-3-(3-dimethylaminopropyl) carbodiimide (0.233 g, 1.5 mM) and 4-dimethylamino pyridine (0.183 g, 1.5 mM). The mixture was reacted at room temperature under stirring for 36 hours, followed by filtration to remove the solid impurity. The filtrate was further reacted with the β-cyclodextrin-modified biguanidine functional molecule (1.92 g, 1.5 mM) in 8 mL of *N,N*-dimethylformamide. After reacting at 60 ^{o}C for 24 hours under vigorous stirring, the

reactive solution was filtrated and then removed the organic solvent by reduced distillation. The crude product was re-dissolved in methanol/hydrochloric acid solution (pH = 5.0, 1/3, v/v) and then excluded the insoluble precipitation by filtration. The filtrate was dialyzed in semipermeable tube (MWCO = 3.0 kDa) to completely remove the organic phase and unreactive ATP-receptor unit, then obtained the final copolymer (denoted as PHM) by lyophilisation [1.96 g, yield: 83%; $M_{n,GPC}$ = 33.5 kDa, $M_{w,GPC}$ = 38.9 kDa, M_w/M_n = 1.16; ^{1}H NMR (d_6-DMSO): δ (ppm) = 6.80 (s), 6.65 (s), 5.65—5.79 (m), 4.81 (m), 4.43—4.58 (m), 4.30 (s), 4.14 (s), 3.96 (s), 3.52—3.75 (m), 3.2—3.45 (m), 2.82 (s), 2.64 (s), 1.89 (s), 1.32—1.58 (m)].

Vesicles loaded with apolar-sensitive DPH probe. DPH was initially dissolved in THF to obtain a 1.0 mM stock solution and then 2.0 μL aliquot of this stock solution was added to a 1.0 mL cuvette containing copolymer solution in THF. The mixture was dropwise into deionized water to form polymer assemblies and removed the unloaded fluorescent dyes by dialysis three times, finally obtaining a DPH-loaded polymer micellar solution.

Co-encapsulation of $NP_{PEI\text{-}x}$ into vesicles. For the encapsulation with dye-modified $NP_{PEI\text{-}x}$ (x = 5, 15 and 25) together into the vesicle lumen, equal molar amount of $NP_{PEI\text{-}5}$ (0.1 mg), $NP_{PEI\text{-}15}$ (0.3 mg) and $NP_{PEI\text{-}5}$ (0.5 mg) in *N,N*-dimethylformamide solution (0.5 mL) were added into the PHM in aqueous solution (0.40 mg/mL). The unloaded $NP_{PEI\text{-}x}$ was removed by dialysis tube (MWCO = 50 kDa). From UV-Vis spectra to monitor the unloaded $NP_{PEI\text{-}x}$ we can know that 0.052 mg of $NP_{PEI\text{-}5}$, 0.137 mg of $NP_{PEI\text{-}15}$, and 0.191 mg of $NP_{PEI\text{-}25}$ were enclosed in the vesicles for subsequent experiments. Dye-modified silica NPs with different sizes or protein blends were encapsulated by a similar way.

Methods. TEM images were measured on a FEI Tecnai G2-F20 S-TWIN instrument at 80 kV accelerating voltage. The specimens were prepared by drop-casting polymer aggregate solutions (10 μL) onto carbon coated copper-grid and freeze-drying before observation. Optical and fluorescent photographs were monitored by Nikon-C2$^+$ laser scanning confocal microscope. ^{1}H, ^{13}C and ^{31}P NMR spectra of functional molecules and polymer sample were obtained by a Bruker AVANCE III HD-400 (400 MHz) spectrometer. The NMR solvent was chosen to be d_6-DMSO according to the experiments. ITC experiments were conducted using a Microcal VP-ITC system at 293.15 K. The concentrations of the model ATP receptor was fixed at 1.0 μM for the addition of ligand solutions (ATP, AMP or Pi is 20 μM in HEPES

buffer). The DLS and surface zeta potential measurements of these polymer aggregates under different conditions were conducted on a Malvern Zetasizer-Nano ZSE instrument. The fluorescence measurements were performed on an Agilent Cary-Eclipse spectrophotometer equipped with a thermostat cell holder. The UV-Vis absorption spectra were recorded on an Agilent Cary-60 spectroscopy. Molecular weights of the polymer samples were performed on a Waters 1515 HPLC system.

2. Supporting Results and Characterization.

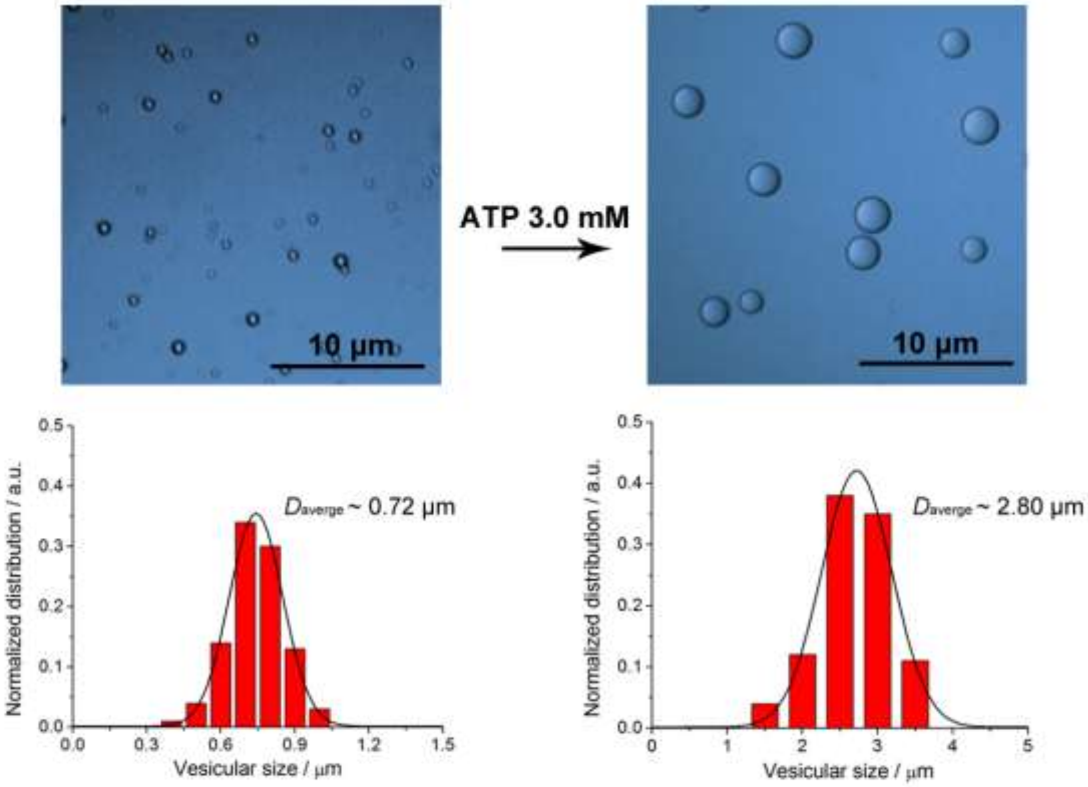

Figure S1. Optical image and particle-size auto-statistics of the PHM vesicles before (left panel) and after (right panel) ATP treatment (3.0 mM). Before the ATP stimulus, the initial vesicular size is averagely 0.72 μm. After addition of ATP, the vesicular size has a 4-fold increase up to averagely 2.80 μm.

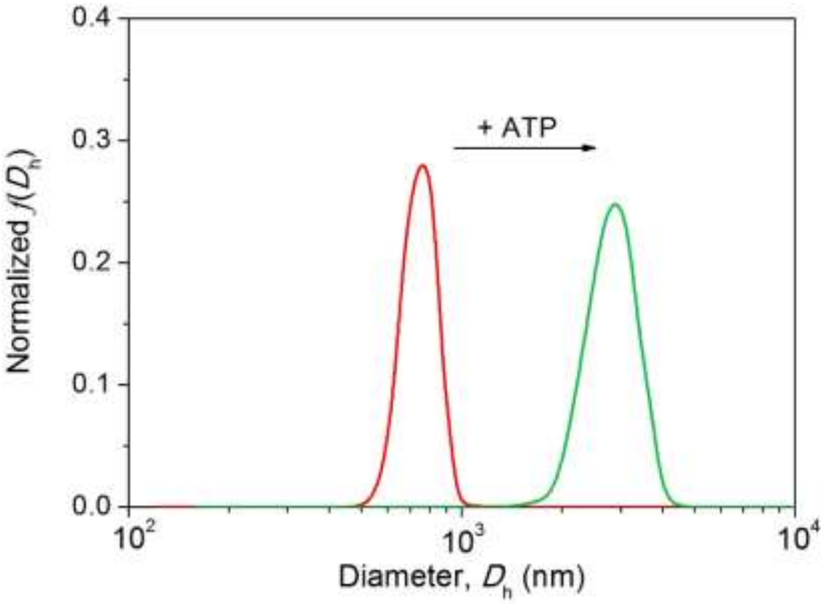

Figure S2. Laser light scattering (LLS) results showing the change of vesicular hydrodynamic diameter (D_h) before and after ATP treatment (3.0 mM). Before addition of ATP, the initial vesicular size is averagely 0.77 µm (PDI = 0.092). After addition of ATP, the vesicular size has a 4-fold increase up to averagely 2.91 µm (PDI = 0.173).

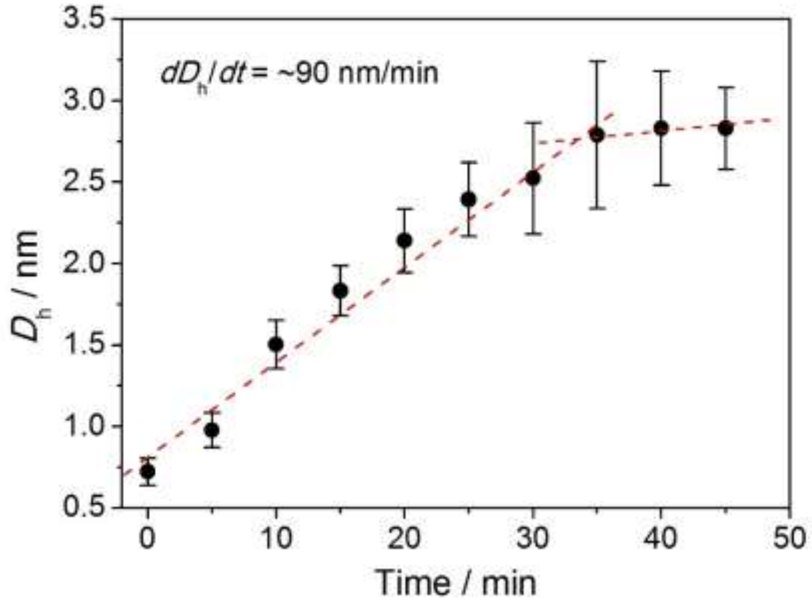

Figure S3. Laser light scattering (LLS) results showing the vesicle size growth rate after treatment with 3.0 mM of ATP. The average size increase growth is around 90 nm min^{-1} within 35 min, and then the vesicle growth stops.

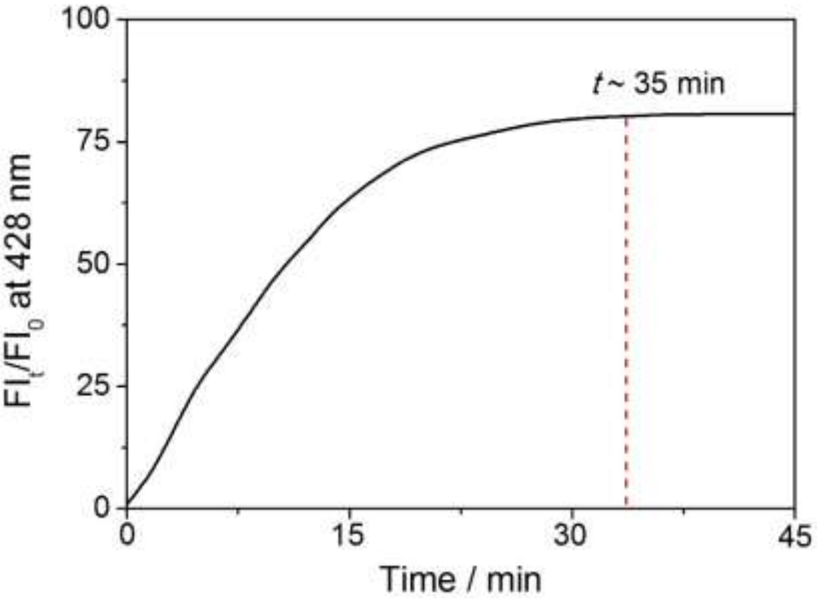

Figure S4. Fluorescent intensity enhancement of DPH-loaded vesicles over time as addition of ATP (3.0 mM). The maximum fluorescent intensity (FI_{max}) appears at around 35 min, which is consistent with that of ATP-regulated vesicular growth by LLS. The DPH characteristic emission is at $\lambda = 428$ nm.

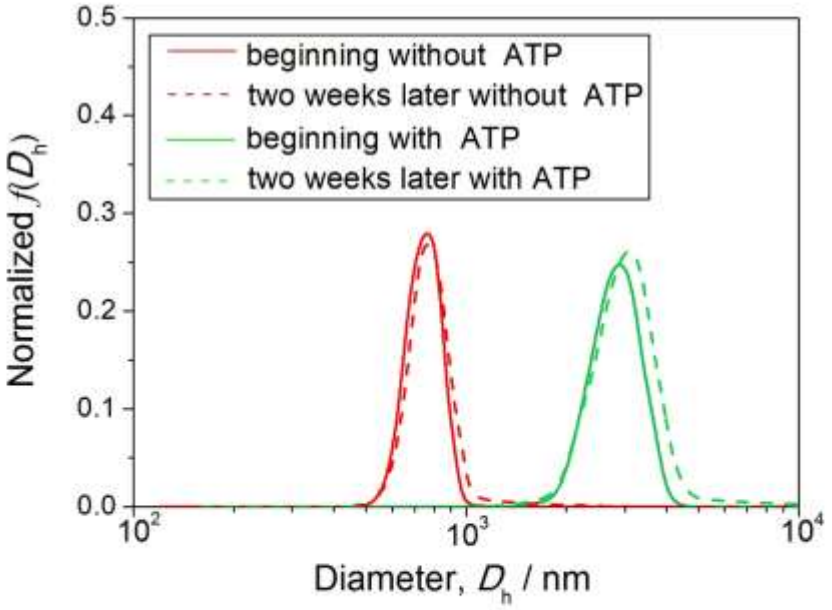

Figure S5. LLS monitoring the invariance of vesicular size in different conditions. The PHM polymer vesicles without any stimuli show stable size at the beginning (red curve, D_h = 0.77 µm, PDI = 0.092) and after two weeks incubation (red dash line, D_h = 0.78 µm, PDI = 0.087); The vesicles that are added 3.0 mM of ATP still show stable size at the beginning (green curve, D_h = 2.91 µm, PDI = 0.172) and after two weeks incubation (green dash curve, D_h = 3.09 µm, PDI = 0.159).

Table S1. The isothermal titration calorimetry (ITC) results between the ligands with different structures and the model ATP-receptor unit showing the binding affinity at 293 K.

Ligand[a]	Receptor[b]	K_a[c] (L mol^{-1})	n[d]	ΔH[e] (kJ mol^{-1})	ΔS (J mol^{-1} K^{-1})	ΔG (kJ mol^{-1})
ATP[f]	ATP receptor	7.52×10^7	0.92	-42.1	7.21	-44.2
Pi[g]	ATP receptor	6.50×10^1	0.38	-8.41	6.40	-8.60
AMP	ATP receptor	4.41×10^2	0.60	-10.65	14.90	-15.1

[a]The ligand are ATP and its hydrolytic products Pi and AMP, whose concentration is at 1.0 mM. [b]The receptor is the model ATP-receptor unit (50 μM), which is comprised of β-cyclodextrin with a biguanidine tail (as following blue structure). [c]The association constants (K_a) are recorded at 293K. [d]The binding-site number (n) represents the stoichiometric ratio between the ligand and the receptor. [e]The mole enthalpy (ΔH), entropy (ΔS) and Gibbs free energy (ΔG) reflect the thermodynamic effect during the ligand-receptor interactions. [f]The strong binding affinity between ATP and ATP-receptor (K_a =7.52×10^7 L mol^{-1}), indicating their tight interaction. [g]The extreme weak binding affinity between either Pi or AMP and ATP-receptor ($K_a < 10^2$ L mol^{-1}), indicating their nearly no interactions (Figure a and b).

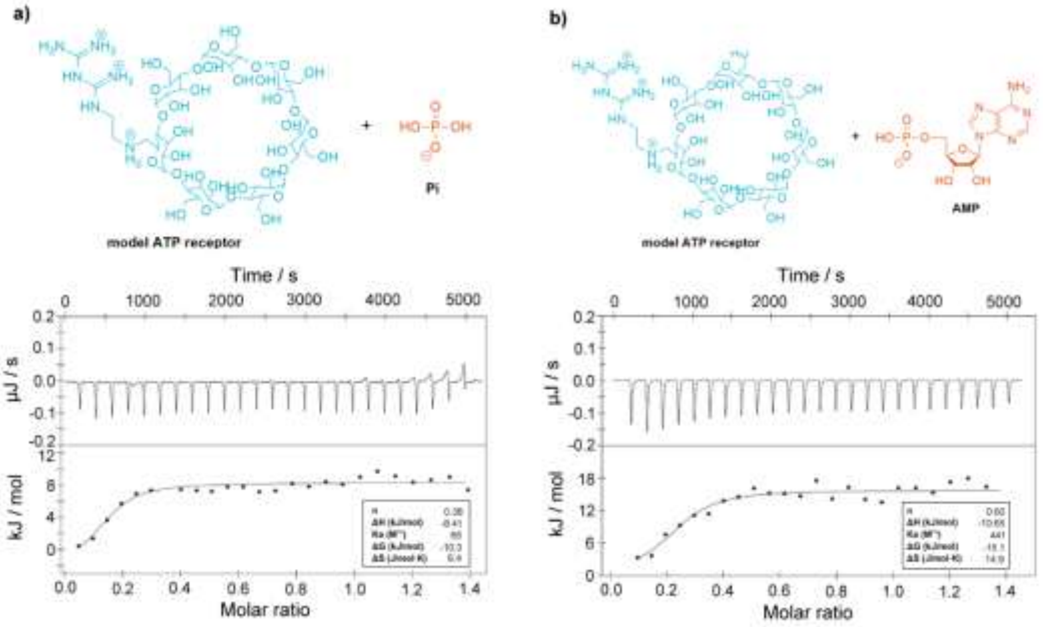

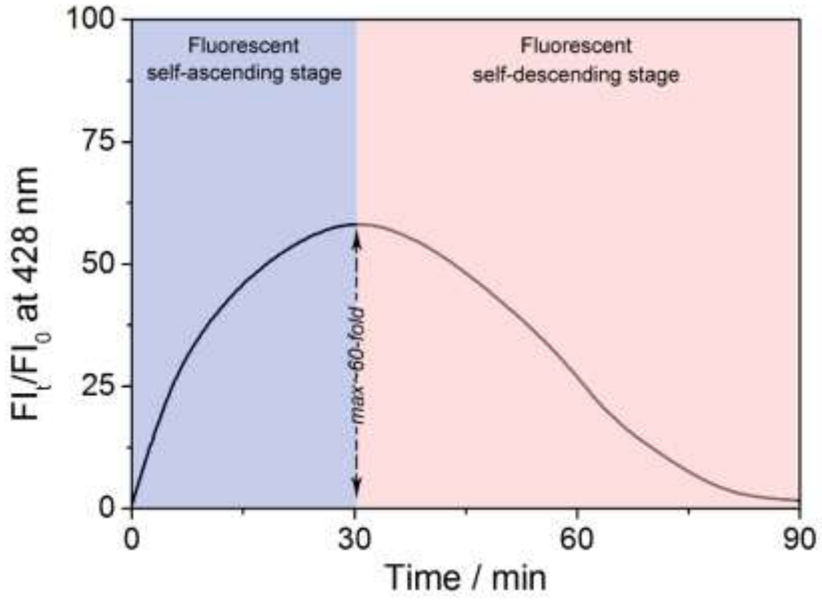

Figure S6. Fluorescent spectrum monitoring the automatic fluorescent regulation of ATP-fuelled polymer vesicles under pretreated with potato apyrase (20 U mL^{-1}). The vesicles are loaded the DPH fluorescent probes. The fluorescent self-regulated process divided into two stages: in the first 30 min, the fluorescence intensity produces a self-ascending to the maximum value of 60-fold enhancement; in the second stage (30→90 min), the fluorescence intensity gives a self-descending back to initial state. This automatic recovery corresponds to the vesicle expansion -contraction process.

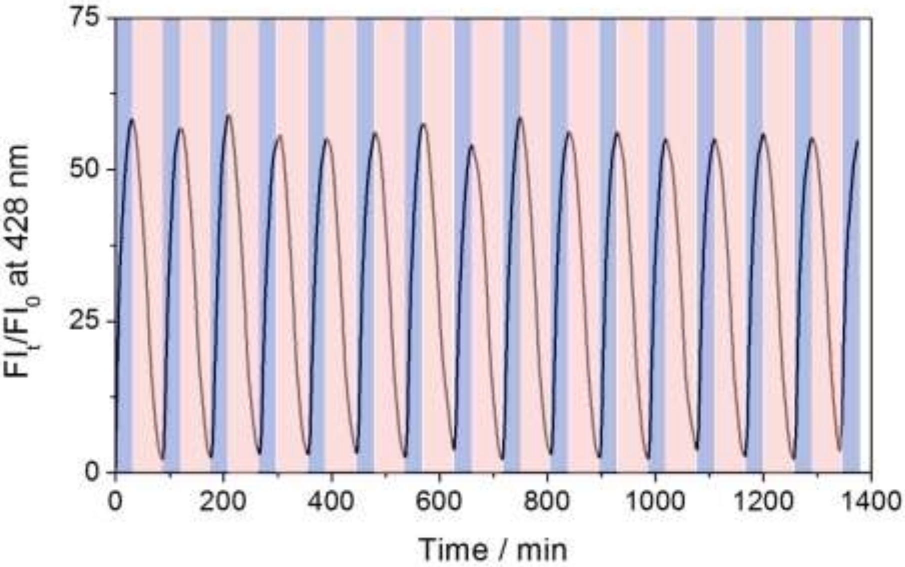

Figure S7. Fluorescent spectrum monitoring the ATP-fuelled periodic oscillating phenomenon of vesicular fluorescence. The PHM vesicles are pretreated with 20 U mL^{-1} potato apyrase and encapsulated with DPH fluorescent probe. Each fluorescent period can be divided into two distinct stages (self-ascending and self-descending stage) and the period time is ca. 90 min (self-ascending time: ~30 min; self-descending time: ~60 min). New batch of ATP can re-initiate this cycle as the fluorescent signal returns back to minimum value.

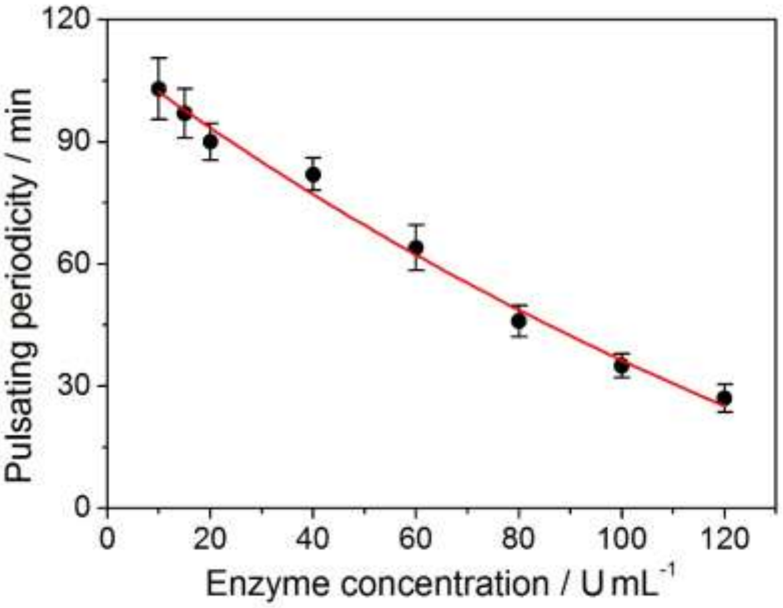

Figure S8. Enzyme concentration plotted against the vesicle pulsating periodicity. ATP concentration is fixed at 3.0 mM.

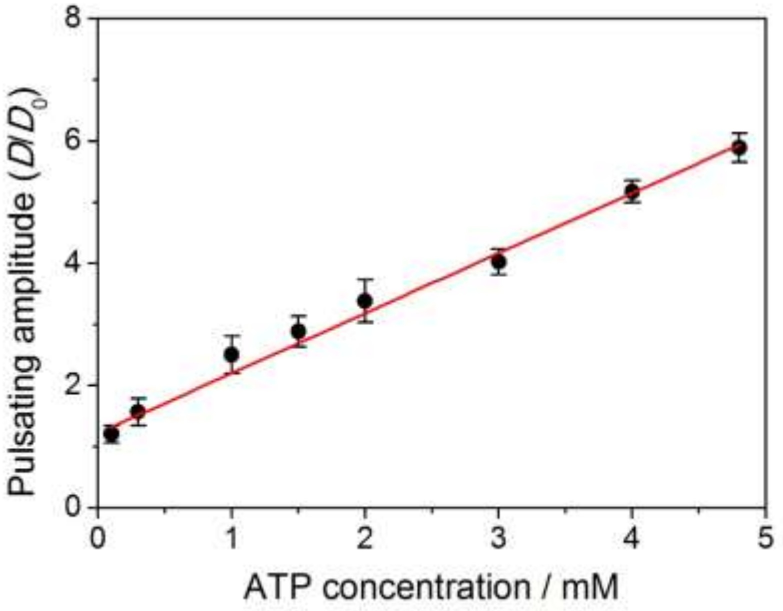

Figure S9. ATP concentration plotted against the vesicle pulsating amplitude (D/D_0). Enzyme concentration is fixed at 20 U mL^{-1}.

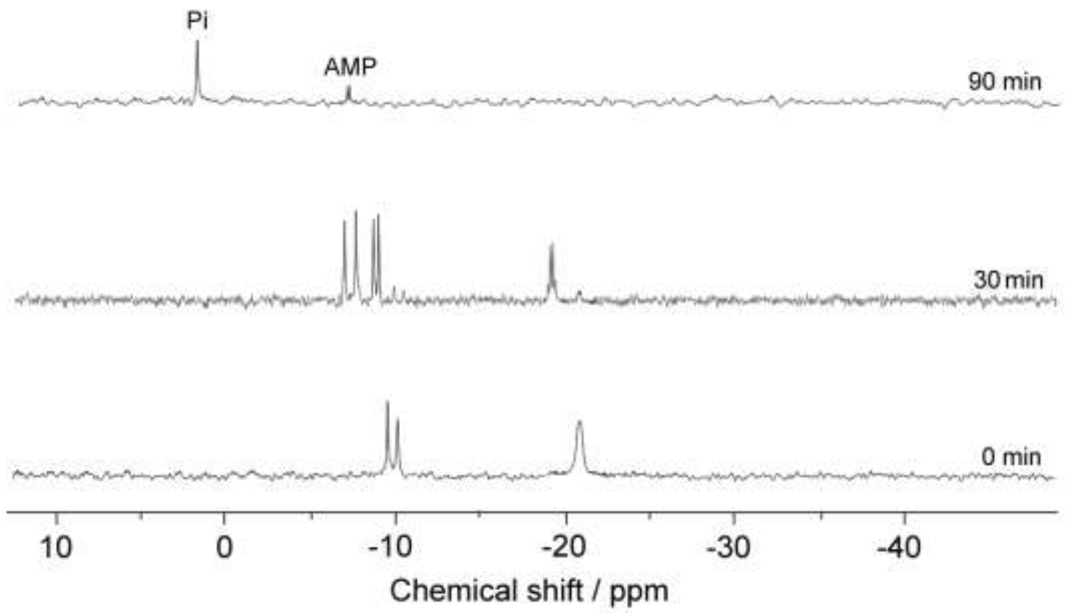

Figure S10. The ^{31}P NMR spectra change showing the association and dissociation of ATP/polymer supramolecular complexes over time. Upon addition of ATP (3.0 mM) to the initial polymer micelles with the enzyme (20 U mL^{-1}), there appeared three typical ATP phosphorus signals at δ = -9.5 (γ-P), -10.9 (α-P), and -21.3 (β-P) ppm (0 min, bottom panel). When the vesicles expanded to maximum size (30 min), the individual ATP signals were strongly depressed whereas three groups of down-shift splitting signals at δ = -7.5 (d, $^3J_{\beta,\gamma}$ = 18.4 Hz), -8.6 (d, $^3J_{\alpha,\beta}$ = 10.5 Hz), and -19.3 (dd) ppm were enhanced, indicating ATP bound to the polymer micelles. After the micelles shrank autonomously to the minimum size (after 90 min), these phosphorus signals ascribed to ATP/polymer complex completely vanished, but a new signal ascribed to Pi (δ = +1.2 ppm) and AMP (δ = -6.3 ppm) species appeared. It indicates that the ATP was decomposed into AMP and Pi by the enzyme.

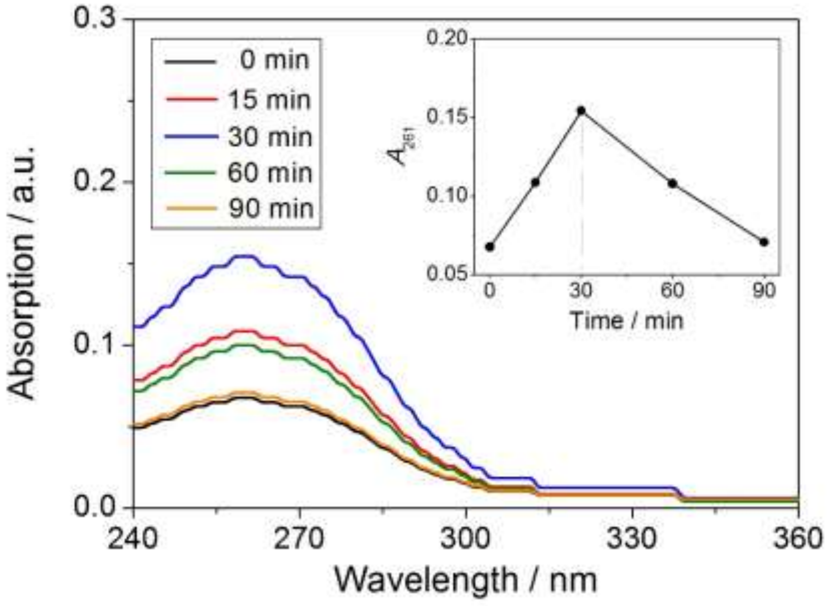

Figure S11. The UV-Vis spectra change as a function of vesicular pulsating motion over time. It has been demonstrated that the vesicular pulsation can be divided into expansive and contractive stages, and the total periodicity is 90 min (vesicle expansion: 0→30 min; contraction: 30→90 min). It is also known that if the ATP molecules associate with the giant vesicles, the characteristic absorption band of ATP (261 nm) can be enhanced; on the contrary, if the ATP dissociates, this absorption can be depressed. UV-Vis spectra change (the change at 261 nm, inset) first displayed a dramatic increase to a maximum value during the first 30 min, corresponding to the vesicular expansive stage, which indicates the ATPs were captured by the copolymers; subsequently, the UV-Vis spectra showed a reversible decrease to a minimum value in 60 min, corresponding to the vesicular contractive stage, which indicates the ATPs were released from the copolymers.

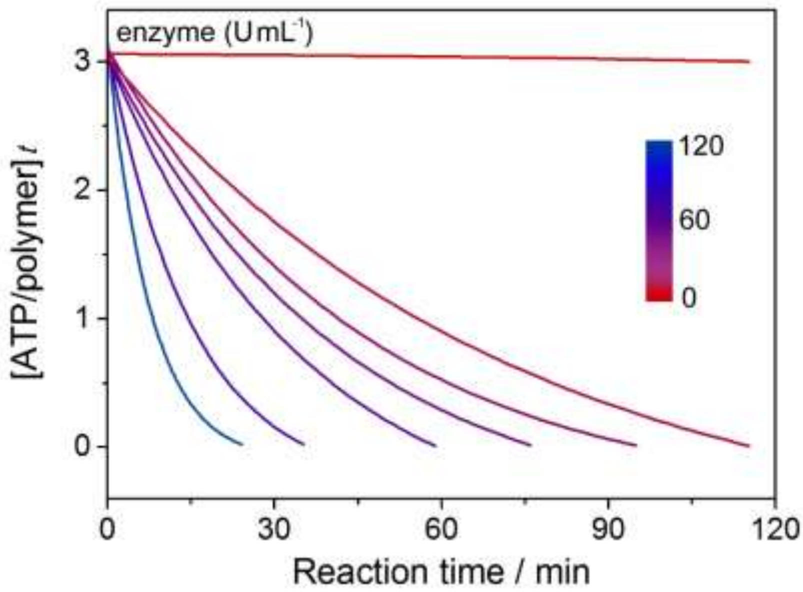

Figure S12. The concentration change of ATP/polymer complex as a function of enzymatic reaction time reflecting the backward dissociation rate under a variety of enzyme stimulus level (0, 20, 40, 60, 80, 100 and 120 U mL^{-1}). The result showed that with the increase of enzyme level, the backward reaction rate is increasingly speeded up (from +∞ to 28 min, 0 U mL^{-1} enzyme: +∞; 20 U mL^{-1}: 115 min; 40 U mL^{-1}: 98 min; 60 U mL^{-1}: 75 min; 80 U mL^{-1}: 58 min; 100 U mL^{-1}: 34 min and 120 U mL^{-1}: 28 min, respectively). ATP concentration is fixed at 3.0 mM in this experiment.

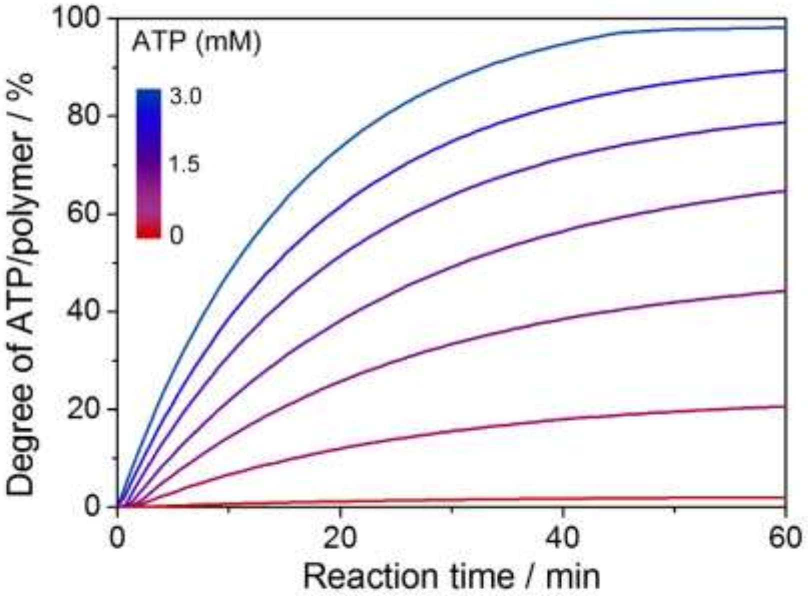

Figure S13. The change of the degree of ATP/polymer complex as a function of association time reflecting the forward association rate under a variety of ATP stimulus level (0, 0.5, 1.0, 1.5, 2.0, 2.5 and 3.0 mM, respectively). The result showed that with the increase of ATP level, the degree of ATP/polymer complex is increased gradually (from <3% to 97%, 0 mM ATP: <3%; 0.5 mM: 19.4%; 1.0 mM: 41.5%; 1.5 mM: 19.4%; 2.0 mM: 63.7%; 2.5 mM: 87.8% and 3.0 mM: 97.1%, respectively).

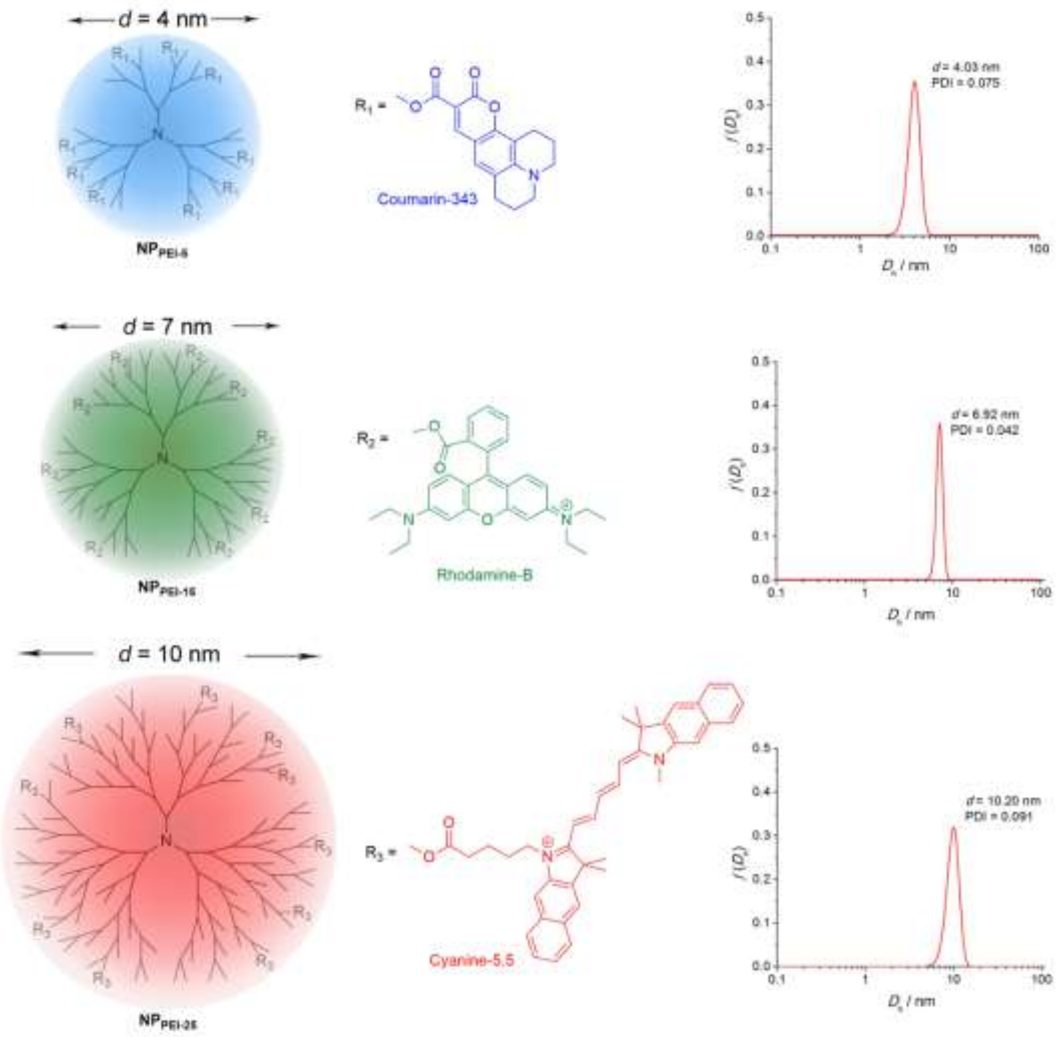

Figure S14. The different sizes of near-monodispersed hyper-branched poly(ethylene imide) (PEI) nanoparticles with different fluorescent labels at the periphery of these polymers: NP_{PEI-5} denotes the 5 kDa of hyper-branched PEI with coumarin-343 label (λ_{em} = 464 nm; nanoparticle diameter, $d \approx 4$ nm, PDI= 0.075; top panel); NP_{PEI-15} denotes the 15 kDa of hyper-branched PEI with rhodamine-B label (λ_{em} = 565 nm; $d \approx 7$ nm, PDI = 0.042; middle panel); NP_{PEI-25} denotes the 25 kDa of PEI with cyanine-5.5 label (λ_{em} = 707 nm; $d \approx 10$ nm, PDI = 0.091; bottom panel).

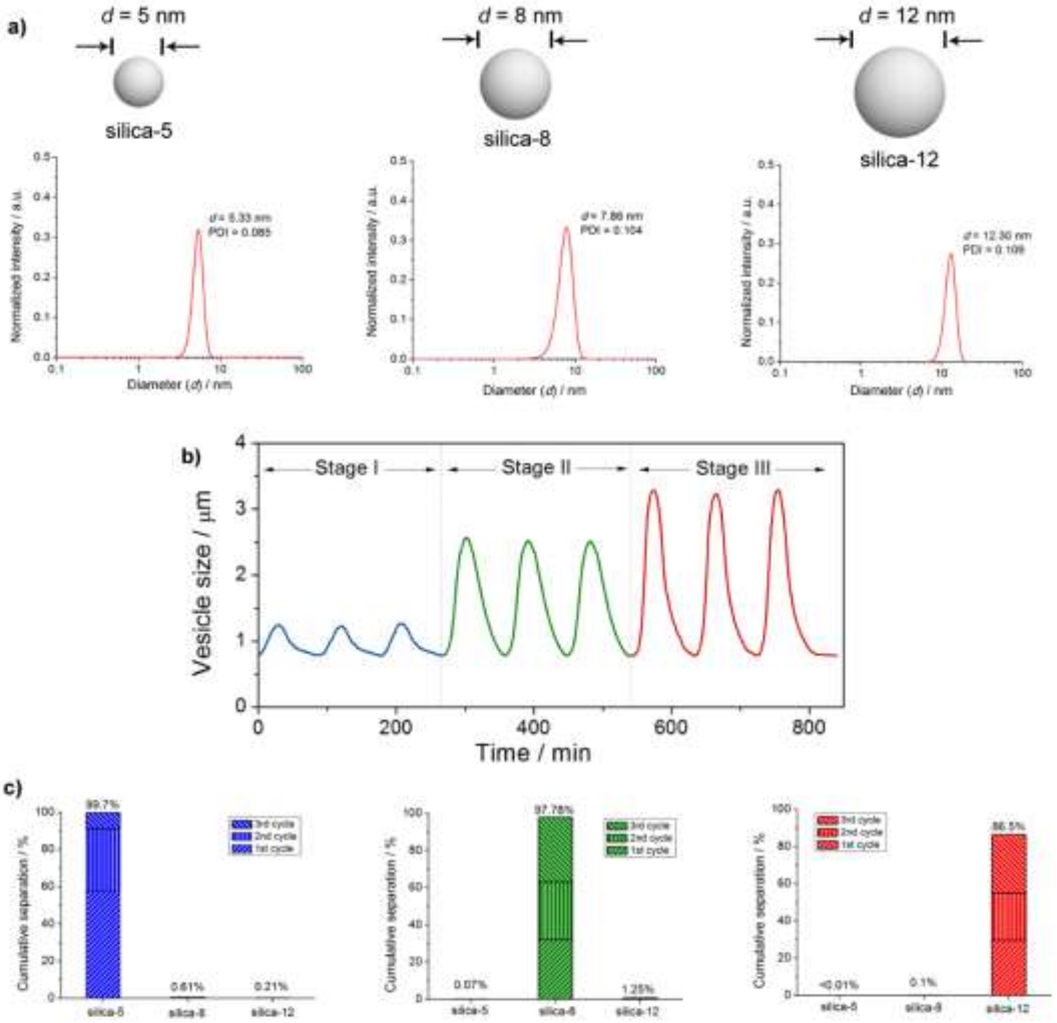

Figure S15. (**a**) DLS results showing the diameter (*d*) of different silica nanoparticles (silica-5 conjugated with coumarin-343 labels, $d \approx 5$ nm, left panel; silica-8 with rhodamine-B labels, $d \approx 8$ nm, middle panel; silica-12 with cyanine-5.5 labels, $d \approx 12$ nm, right panel). (**b**) Programmed vesicular pulsation process with three distinct stages (Stage I: blue curve, 0.5 mM ATP fuel leading to 2.0-fold size jump; Stage II: green curve, 1.5 mM ATP fuel leading to 3.0-fold size jump; Stage III: red curve, 4.0 mM ATP fuel leading to 4.8-fold size jump). Each stage contains three pulsating cycles. (**c**) Separation efficiency with respect to the different sizes of silica nanoparticles in the three stages: Stage I for selectively sieving silica-5 with 99.7% sieving fraction (**c**), Stage II for selectively sieving silica-8 with 97.8% sieving fraction (**d**), and Stage III selectively sieving silica-12 with 86.5% sieving fraction (**e**).

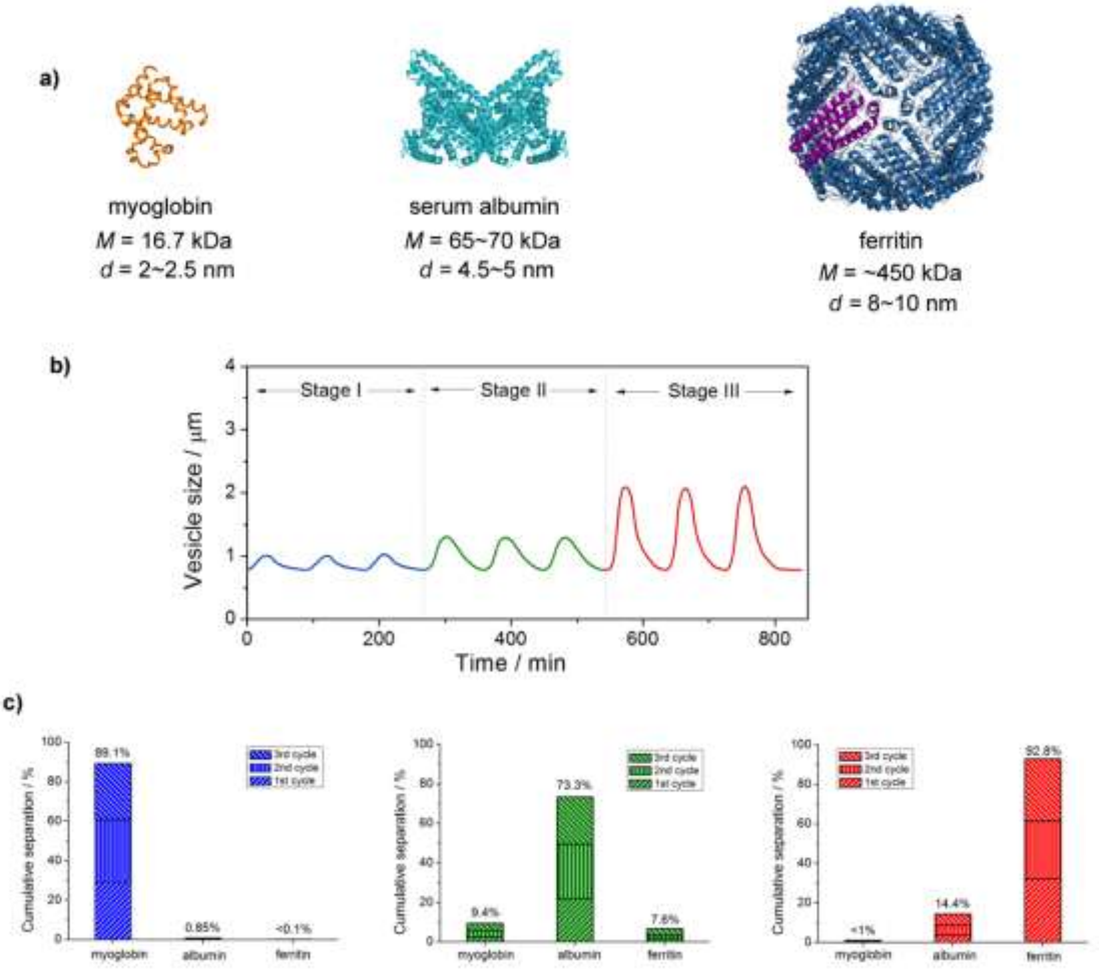

Figure S16. (**a**) The type and diameter (*d*) of multiple protein blend (myoglobin surface modified with coumarin-343, *d* = 2~2.5 nm, left panel; serum albumin surface modified with rhodamine-B, *d* = 4~4.5 nm, middle panel; ferritin surface modified with cyanine-5.5, *d* = 8~10 nm, right panel). (**b**) Programmed vesicular pulsation process with three distinct stages (Stage I: blue curve, 0.15 mM ATP fuel leading to 1.2-fold size jump; Stage II: green curve, 0.3 mM ATP fuel leading to 1.6-fold size jump; Stage III: red curve, 1.2 mM ATP fuel leading to 3.0-fold size jump). Each stage contains three pulsating cycles. (**c**) Separation efficiency with respect to the different sizes of protein nanoparticles in the three stages: Stage I for selectively sieving myoglobin with 89.1% sieving fraction (**c**), Stage II for selectively sieving serum albumin with 73.7% sieving fraction (**d**), and Stage III selectively sieving ferritin with 92.8% sieving fraction (**e**).
